# Assessment of the Health Status of Mussels *Mytilus galloprovincialis* Along the Campania Coastal Areas: A Multidisciplinary Approach

**DOI:** 10.3389/fphys.2018.00683

**Published:** 2018-06-12

**Authors:** Francesca Carella, Serena Aceto, Olga Mangoni, Maria Pina Mollica, Gina Cavaliere, Giovanna Trinchese, Francesco Aniello, Gionata De Vico

**Affiliations:** Department of Biology, University of Naples Federico II, Naples, Italy

**Keywords:** biomarkers, environmental stressors, cell damage, mussel, health status, mitochondrial function

## Abstract

The bivalve *Mytilus galloprovincialis* has a broad geographic distribution, represent an important species for the ecology of coastal waters, also constituting a major aquaculture species. In the present work, molecular and tissue biomarkers were examined in mussel populations (*M. galloprovincialis*) located in four different areas of the coastal water of the Campania Region. During an annual life cycle, we analyzed the expression patterns of several genes commonly used to estimate cellular stress response and damage, namely *p53*, *p63*, *HSP70*, *MT-10*, and *MT-20*, related tissue lesions (pathogens, inflammations, digestive tubules damage), oxidative stress indicators (H_2_O_2_, SOD specific activity) and associated environmental data. The computed Principal Component Analysis showed that the areas were discernible based on the environmental data and biomarker results. About animal health status, mussels from Gulf of Pozzuoli and Naples’s harbor did show a thinnest epithelial cell of digestive tubules compared to mussels sampled from other sampling sites; moreover, high prevalence of cases of intersex in three of the examinated areas were observed. The presence of a potential zoonotic pathogen (*Nocardia crassostreae*) was identified, appearing as an important possible emerging disease. We also reported the OIE notifiable protozoa *Marteilia refringens* in three areas out of four. The likely impact of both observed pathogens on the mussel health and shellfish aquaculture needs to be urgently addressed. Results are discussed considering animal histopathological health parameters and biological effects.

## Introduction

Marine animals lives in polluted coastal waters nearness anthropized areas are more exposed to a multiple toxic substances of domestic and industrial origin. Increasing social apprehension about the environmental quality and vulnerability of biodiversity of the marine coastal areas have been observed in recent years, both on a global and local scale (de Sherbinin et al., 2007; Beaugrand et al., 2015; Ramiìrez et al., 2017). Historically, bivalve mollusc *Mytilus galloprovincialis* has an important tradition in Italy as both food and income source from natural banks and farming production (FAO, 2015). Relatively little is known about mussels health status in the area and the effect of pathogens, source of stress and parasites in this region (Carella et al., 2010, 2013a,b; Trinchella et al., 2013).

Lately, several studies have highlighted the importance of biomarkers integrated approach to describe the health status of animal organisms in order to understand the impact of climate change and human activities (Salihoglu et al., 2013; Hook et al., 2014; Mangoni et al., 2016). Mussels, as all the filter feeders, are exposed to a variety of natural stressors that, along with genetic differences in susceptibility to stress, result in an increased variability of molecular and tissue responses (Marigomez et al., 2013). The use of battery of biomarkers can result, for all these reasons, necessary and useful in environments where complex combinations of environmental factor can cooperate in a site. A variety of contaminant/stress-related biomarkers have been suggested to be monitored for an integrated assessment of contaminants, as well as their effects in different seas (Lehtonen et al., 2014). These include tissue end-points, represented by histopathological alterations at a bivalve digestive gland, neoplastic and inflammatory lesions, levels of metallothionein gene expression (Knapen et al., 2007; Gagné et al., 2007), superoxide dismutase (SOD) specific activity and mitochondrial function (Abele et al., 2007; Cravo et al., 2009).

The coastline of the Campania Region extends for 450 km on the Eastern Tyrrhenian Sea margin and forms three main bays, comprehend from North to South, the Gulfs of Gaeta, Naples and Salerno. The region shoreline is densely populated and characterized by the presence of many industrial and harbor activities, commercial discharges as well as includes some protected or pristine areas (Imperato et al., 2003; Altavista et al., 2004; Trifuoggi et al., 2017). Consequently, the coastal water presents distinct trophic characteristics depending on natural and artificial terrestrial inputs (e.g., river runoff, exchanges with adjacent basins), coast orography and coastal circulation. For all the above reasons, in the area it is possible to detect highly distinct eutrophic characteristics, typical of heavily impacted marine waters, as oligotrophic characteristics that are typical of the open sea system of the Tyrrhenian Sea and, more generally, of the entire Mediterranean (Ribera d’Alcalà et al., 2004; Pugnetti et al., 2006; Mangoni et al., 2016).

In order to define the health status of the mussel (*M. galloprovincialis*) population of the area, we carried out a multidisciplinary study reporting animal tissue lesions (inflammation, digestive tubules damage) expression patterns of the genes *p53, p63, HSP70*, *MT-10*, and *MT-20*, oxidative stress indicators (H_2_O_2_, SOD) and environmental data (temperature, inorganic nutrient and chlorophyll-a) during the annual life cycle (Lauenstein, 2012) of *M. galloprovincialis*. Four different areas in coastal water of the Campania Region were sampled to define the local mussel health status. The aim is to establish possible biological responses associated with potential sources of stress and to determine spatial and seasonal trends. This is the first study of systematic biomonitoring for a whole year in the Tyrrhenian Sea, using as bio-indicator the indigenous mussels *M. galloprovincialis* and the seasonal variations of the above parameters.

## Materials and Methods

### Sampling Sites and Sample Collection

The study area consisted of four distinct coastal stations along the marine coastal water of the Campania region (southern Italy). The sites comprehended one farm in the southeast part of the sampling area, named Torre del Greco (TG) and three natural banks in the northern part of the coastline: Litorale Domitio (LD), Pozzuoli (PO), and Naples harbor (PN). In particular, the Naples’s harbor an important commercial and touristic harbor of the Mediterranean Sea, receives high load of municipal discharges (both untreated and with only primary management) of 1.5 million people. In addition, several industrial activities, port activities (shipbuilding, commercial and tourist transaction) and commercial discharges, takes place in the area (Sprovieri et al., 2007). About the Gulf of Pozzuoli, over the years it has been intensely impacted by different anthropic activities from the brownfield-like the ILVA of Bagnoli, the second largest integrated steel plant in Italy now under remediation by a Government project (Albanese et al., 2010; Arienzo et al., 2017). The collection site was at the level of the Pier of Bagnoli, in the middle of the area. Finally, Litorale Domitio is located in the north-western coast of the Campania region and is affected by industrial activity (area of production of buffalo Mozzarella), extensive agriculture and other human activities. This district lies between the Patria lake and the River Volturno on an area includes both housing estates and tourist infra-structures (Iterar et al., 2012). The mussels were collected on a floating rope of a temporarily disabled fish cage. **Table 1** reports the GPS coordinates for each site and the time course of the samplings. Each sampling consisted of a total of 60 specimens of *M. galloprovincialis* (5.2 ± 0.8 cm shell length), used for the different analyses. Mussels were transferred alive and kept cool in an insulated sealed container for transit to the laboratory, in order to limit any animal stress and subsequent analysis artifact that may result from the sampling and transport process. Animals were processed for histopathology, gene expression (*p53, p63, HSP70, MT-10*, and *MT-20*), oxidative stress (H_2_O_2_, SOD), body composition and body energy. Temperature, inorganic nutrient and chlorophyll-a (Chl-a) data were obtained at the surface to assessing the trophic state of different systems at the time of mussel sampling.

**Table 1:** Collection sites and time course of the samplings.

Site name	Abbreviation	Latitude	Longitude	Characteristics	Collection date (dd/mm/yyyy)	Symbol/season
Litorale Domizio (Torregaveta)	LD	40°48′31.50″N	14°2′25.56″E	Natural population	01/03/2012 15/05/2012 12/07/2012 11/10/2012	(a) Winter (b) Spring (c) Summer (d) Autumn
Pozzuoli (Miseno)	PO	40°46′50.40″N	14°5′46.96″E	Natural population	01/03/2012 15/05/2012 12/07/2012 11/10/2012	(a) Winter (b) Spring (c) Summer (d) Autumn
Porto di Napoli	PN	40°50′35.40″N	14°16′18.40″E	Natural population	12/12/2011 17/05/2012 09/07/2012 11/10/2012	(a) Winter (b) Spring (c) Summer (d) Autumn
Torre del Greco	TG	40°47′6.39″N	14°21′38.73″E	Farmed population	07/12/2011 17/05/2012 09/07/2012 17/10/2010	(a) Winter (b) Spring (c) Summer (d) Autumn

In addition, 60 specimens of *M. galloprovincialis* were acclimated at the *Marine Resources for Research Service* (Ma.Re.R) animal rearing facility of the Stazione Zoologica Anton Dohrn (SZN). The animals were placed into open–meshed trays and suspended in a flow-through system with filtered (0.35 μm) seawater (temperature: 18°C, salinity: 33% 0, 1 L/animal) and natural photoperiod for 60 days. The water quality parameters (ammonium, nitrate, oxygen, and pH) was checked every day. During this period, the mussels were fed with a mixture of *Isochrysis galbana*, *Tetraselmis suecica*, and *Skeletonema costatum* in a continuous algae supply over a period of 24 h of 150 cells μl^-1^. The animals were used as control for gene expression analysis.

### Pathological Evaluation

Thirty mussels for area for season were processed for routine histopathology. Animal valves were measured. From each animal a transverse section including, mantle, digestive gland, gills and foot were fixed in Davidson’s solution for at least 48 h at room temperature. Another section comprehending heart and kidney was also taken. Subsequently, cuts were embedded in paraffin blocks, sectioned (3–5 μM thick) at rotary microtome, stained with routine haematoxylin and eosin (H&E) and observed at light microscopy. Additional stains, such as V.O.F. for *Marteilia* sp. (Gutiérrez, 1977), Masson’s Trichrome and Schmorl for lipofuscins and Alcian Blue-Periodic Acid/Schiff’s (AB-PAS) for polysaccharides (Mazzi, 1977; Howard and Smith, 1983), were also used. All micrographs were captured using a Nikon DS-Fi1 video camera mounted on a Nikon 50i microscope, connected to a computer. After staining, the slides were examined at light microscope for histopathological diagnosis. Blind-coded labels were used in order to avoid operator’s subjectivity. For each sampling, the prevalence of pathological changes (inflammations, tumors, change of sex), regressive phenomena (digestive tubule atrophy, tissue necrosis and lipofuscin accumulation), pathogens (Prokaryote *Nocardia crassostreae*, Protozoa *Martelia refringens*, Fungi *Steinhausia mytilovum* and Helminths Trematodes) and organism reported by literature as symbionts (Idrozoa *Eugymnanthea inquilina*, Protozoa Ciliates *Ancystrum mytili* and Platelmintes Turbellarian *Urostoma* sp.) was evaluated and singularly considered (Carella et al., 2015a), according to Kim et al. (2006).

Defensive phenomena were recorded and evaluated as specific response to pathogens or as unspecific reactions. For each population sample, prevalence of inflammatory lesions was documented and morphologically classified as percentage of Infiltrates, Nodules and Capsules (De Vico and Carella, 2012). Moreover, in the observed samples, a semi-quantitative measure of haemic reaction was also assessed. In particular, each mussel specimen was scored using arbitrary scales for intensity of inflammatory lesions, by visual examination of a single histological section for each case at 40X of magnification. Scales ranged from 1 (25% of inflammation extended to digestive system) to 4 (heavy inflammatory lesions widespread >90% of the digestive system), with the scores 1 and 2 as intermediate levels (Carella et al., 2015b).

For the regressive changes, a semi-quantitative evaluation of the percentage of atrophic digestive tubules (Epithelial Thickness–ET) in the whole tissue section was scored as reported by Kim et al. (2006).

### Gene Expression Analysis

Total RNA was extracted from digestive gland tissue (50 mg) of six specimens stored in RNA Later (Ambion Inc., Austin, TX, United States) at -20°C from each sampling site using TRIzol reagent (Ambion Inc., Austin, TX, United States) followed by DNase treatment (Carella et al., 2013a). In addition, the digestive gland of two individuals of *M. galloprovincialis* from each sampling site acclimated as described above was used as control tissue. Total RNA was quantified using the spectrophotometer NanoDrop 2000c (ThermoScientific) and its integrity was checked using the Bioanalyzer (Agilent Technologies). One microgram of each RNA per sampling was pooled, resulting in two pools of total RNA from three individuals per sampling. For each pool, three micrograms of total RNA were reverse transcribed using the reverse transcriptase Superscript III (Invitrogen, San Diego, CA, United States) and random hexamers following the conditions previously described (Carella et al., 2013a). Real Time PCR experiments were conducted to measure the relative expression level of the genes *MT-10*, *MT-20*, *p53, p63*, and *HSP70* using the 18S rRNA as endogenous control gene, following the conditions previously described (Aceto et al., 2011). The reactions were conducted in technical triplicates. Primer pairs specific for each gene are listed in **Table 2**. Primers to amplify *MT-10, MT-20* and 18S were previously reported in Aceto et al. (2011); primers to amplify *HSP70* and *p53* were designed using the Primer Express software v.3.0 (Applied Biosystems), based on the sequence of the *HSP70* (accession number AY861684), *p53* (KC545827) and *p63* (MF997537) mRNA of *M. galloprovincialis* present in GenBank. The relative expression levels of the target genes were calculated applying the comparative Ct (ΔΔCt) method, using the digestive gland cDNA of the acclimated animals as the reference sample and the 18S as the endogenous control. Differences in the relative expression levels among the sampled populations were assessed by ANOVA analysis followed by a Games–Howell *post hoc* test.

**Table 2:** Sequence (5′-3′) of the primer pairs used to perform the Real Time PCR experiments.

Gene	Forward	Reverse
*MT-10*	GGGCGCCGACTGTAAATGTTC	CACGTTGAAGGCCCTGTACACC
*MT-20*	TGTGAAAGTGGCTGCGGA	GTACAGCCACATCCACACGC
*p53*	TGGCTGTCGATGATACTGG	TTGATTCCTGGTCGATGTTG
*p63*	CCAATGAGCCAGGAAACATT	GGTTTCATCCTCAGCTTCACT
*Hsp70*	GGACAAGCAACAAAGGATGC	CCACCACCCAAGTCAAAGAT
*18S*	TCGATGGTACGTGATATGCC	CGTTTCTCATGCTCCCTCTC

*Primers for MT-10 and MT-20 are from Aceto et al. (2011).*

### Lipid Extraction and Body Composition

The total lipid content was determined by using the method of Folch et al. (1957). To obtain the lipid fraction, the tissue was collected from 5 different individuals (with 3 replicates), washed with filtered seawater and finely minced. Lastly 0.5 g of tissue was homogenized in 1.1% NaCl and lipids were extracted in chloroform:methanol solution (2:1, v/v).

Energy, water, fat and protein content in animal carcasses were measured according to Iossa et al. (2002).

### Preparation of the Mitochondrial Fraction

After mussels dissection, mantle and gill tissues were removed, pooled and washed in ice-cold medium A (0.25 M sucrose, 5 mM Tris-EDTA, pH 7.4). The tissues were dried, weighted and subsequently homogenized in medium B [0.25 mM sucrose, 0.5 g/L bovine serum albumin (BSA), Tris 24 mM, pH 7.6], in the proportion 11 mL medium B/g of tissue, by Ultraturrax T25 (IKA-Labortechnik) at 14,000 rpm for 1 min. The mitochondrial fraction from gills and mantle was obtained by stepwise centrifugation. The homogenate was centrifuged at 1500 *g* for 10 min, the supernatant was filtered through sterile gauze layers and further centrifuged at 9000 *g* for 12 min. The resulting precipitate was resuspended in medium B and further centrifuged at the same speed for 12 min to obtain the mitochondrial pellet which was resuspended in a small quantity of medium B, to obtain a protein concentration of 10–12 mg/mL determined by Hartree method (Hartree, 1972). All steps were carried out at 0–4°C.

### Evaluation of Mitochondrial Respiratory Activities

Mitochondrial oxygen consumption was measured polarographically with a Clark-type electrode in a 3-ml glass cells, at a temperature of 20°C (Estabrook, 1967). Isolated mitochondria (1 mg/ml) were incubated in a medium containing 0.2 M KCl, 5 μM cytochrome c, 5 mg/mL fatty acid-free BSA, 20 mM Tris–HCl, pH 7.2; 6 mM KH2PO4. The rate of oxygen consumption was assessed in the presence of 3 mM of succinate for complex II and 0.6 mM of ADP. The respiratory control ratio (RCR), calculated as the ratio between State 3 and 4 activities, were determined as defined by Chance and Williams (1955). Polarographic assays of mitochondrial preparations from distinct animal pools were carried out at least in triplicate, according to Pagliarani et al. (1994).

### Determination of Mitochondrial SOD Activity and H_2_O_2_ Release

Superoxide dismutase specific activity was measured spectrophotometrically (550 nm) at 25°C, by monitoring the decrease in the reduction rate of cytochrome c by superoxide radicals, generated by the xanthine–xanthine oxidase system. Mitochondria were incubated in a medium containing 0.1 mM EDTA, 2 mM KCN, 50 mM KH2PO4, pH 7.8, 20 mM cytochrome c, 5 mM xanthyne, and 0.01 U of xanthyne oxidase. One unit of SOD activity is defined as the concentration of enzyme that inhibits cytochrome c reduction by 50% in the presence of xanthine + xanthine oxidase (Flohé and Otting, 1984).

Rate of mitochondrial H_2_O_2_ release was assayed by following the linear increase in fluorescence (excitation at 320 nm, emission at 400 nm) due to the oxidation of homovanillic acid in the presence of horseradish peroxidase by using Jasco fluorometer equipped with a thermostatically controlled cell-holder (30°C). Mitochondrial proteins (0,4 mg) and succinate (0,6 M) were incubated in the solution used for the determination of O2 consumption. Different known concentrations of H_2_O_2_ (from 0.5 μM to 8 μM) were progressively added to establish the standard concentration curve (Barja, 1998).

### Physical and Chemical Data

Temperature data, at the time of mussel collection, was obtained from a Sea Bird Electronic, SBE 19 Plus CTD probe. Surface water samples were collected at the surface from the Niskin bottle at the end of the trophic state of different systems. After careful mixing, subsamples were distributed to determine inorganic nutrient and phytoplankton biomass (in terms of chlorophyll-a).

To determine nutrient concentrations (NO3-, NO2-, NH4+, Si(OH)4, PO43-) samples were collected in 20 mL low-density polyethylene containers and stored at 20°C until further analysis. The analyses were performed using a Flow Sys System autoanalyzer, following the procedure described by Hansen and Grasshoff (1983). 500 mL of seawater were filtered into 25 mm GF/F Whatman filters for phytoplankton biomass. Filters were stored in liquid nitrogen until further analysis. The analyses of chlorophyll-a (Chl-a) and phaeopigments (Phaeo) were carried out according to Jeffrey and Humphrey (1975), with a spectrofluorometer (Spex), which was checked daily with a Chl-a standard solution (from Anacystis nidulans; Sigma).

### Statistical Analysis

The statistical analysis of the data was performed using SPSS 21 (SPSS Inc., Chicago, IL, United States). Normality of data (Kolmogorov–Smirnov’s test) were tested before statistical analysis.

Principal component analysis (PCA) was performed to group the data set into few variables that could explain most of the variance associated to the samples. Pearson correlation coefficients were calculated between gene expression values, biomarker responses, and environmental data.

## Results

Results obtained from all the analyses of the mussels (*M. galloprovincialis*) sampled in the 4 localities along the Campanian coast are given in **Figures 1**–**9** and **Tables 1**–**5**.

**FIGURE 1:**
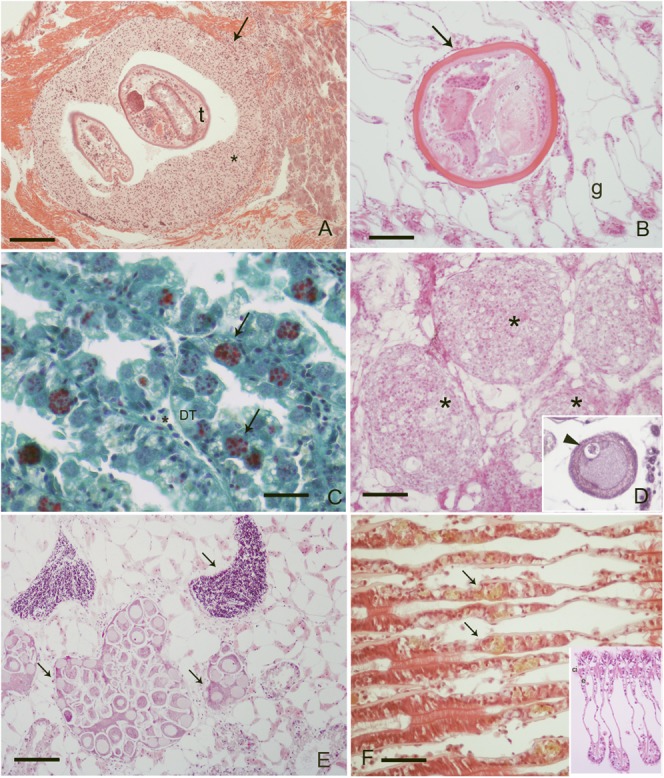
Pathogens and diseases observed during the study: **(A)** Inflammatory capsule (^∗^) surrounding trematodes (t) parasites at mussel foot level, (Scale bar: 100 μM); **(B)** unknown metacercaria at gill level (g) with no immune response from the host (Scale bar: 50 μM); **(C)** *M. refringens* (arrows) in the digestive tubules (DT) of mussels, (Scale bar: 10 μM). V.O.F. Stain; **(D)** nodulation at gondal level in infection by *S. mytilovum* (insert, arrowhead) (Scale bar: 50 μM); **(E)** DSD (*Disorders of Sex Development*) at gonadal level: note the presence of both male and female follicles in the same individual (Scale bar: 100 μM); **(F)** intraepithelial haemocytes (arrows) at gill level containing lipofuscin (arrows), (Scale bar: 50 μM) insert: normal gills; e, epithelia; ci, cilia; ^∗^, haemocytes.

**FIGURE 2:**
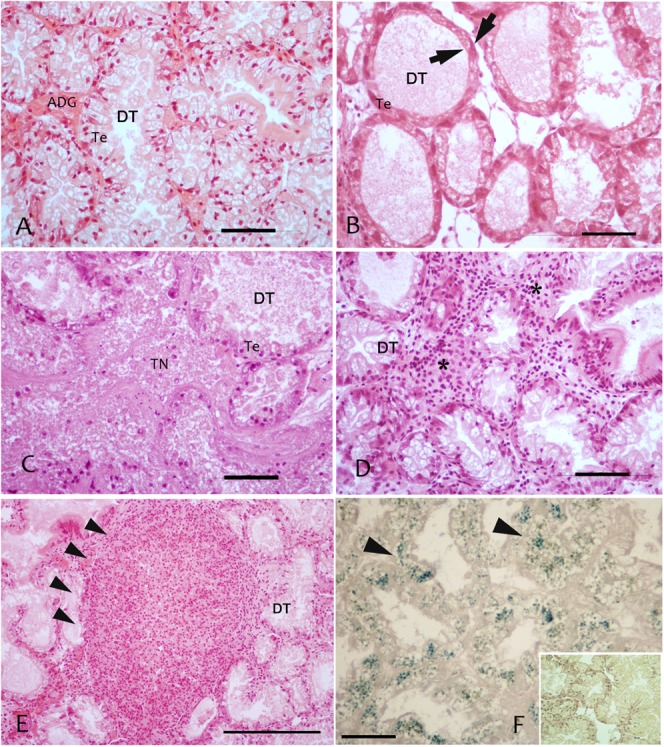
Regressive and inflammatory lesion at digestive gland level observed in the studied areas in sampled mussels *M. galloprovincialis;* **(A)** Digestive tubules (DT), showing a normal adsorbing epithelia (TE); **(B)** Atrophic digestive tubules (DT) with epithelia thinning (arrows) in PN; **(C)** Colliquative Necrosis (TN) of digestive tubules in LD; **(D)** Infiltrative inflammation (^∗^) among digestive tubules; **(E)** Inflammatory nodules (arrowheads) occupying most of the digestive tissue; **(F)** Lipofuscin accumulation in digestive cells of DT underlined by Schmorl staining and visible as yellow pigment in H&E stain (insert). Scale bar: 50 μM. TN, tissue Necrosis; Te, tubule epithelium; DT, digestive tubules; ^∗^, haemocytes.

**FIGURE 3:**
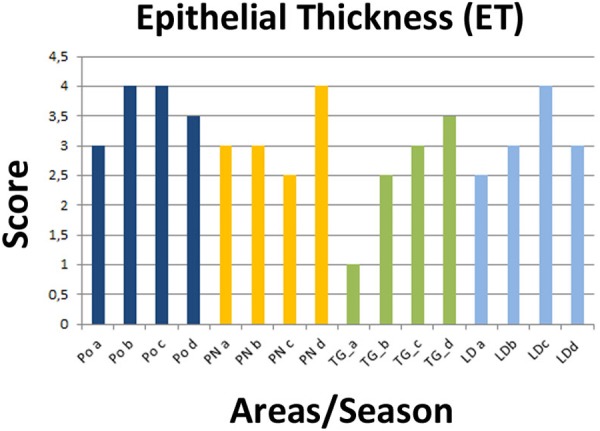
Recorded epithelial thickness (ET) in the sampled areas (PO, PN, TG, LD). Values from 1 to 4 referred to an increased percentage of atrophic digestive tubules as reported in Kim et al. (2006).

**FIGURE 4:**
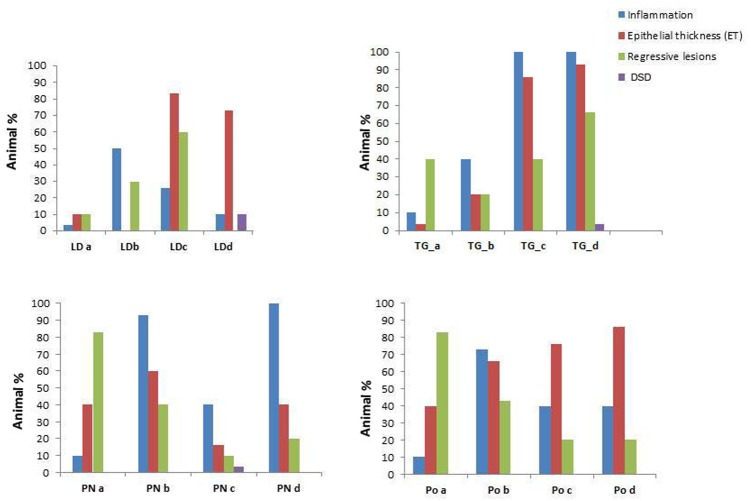
Prevalence of the pathological conditions (inflammation, epithelial thickness-ET, Regressive lesions and Disorsers of Sex Development – DSD) observed in the different tissues in *M. galloprovincialis* collected at the different sites and periods.

**FIGURE 5:**
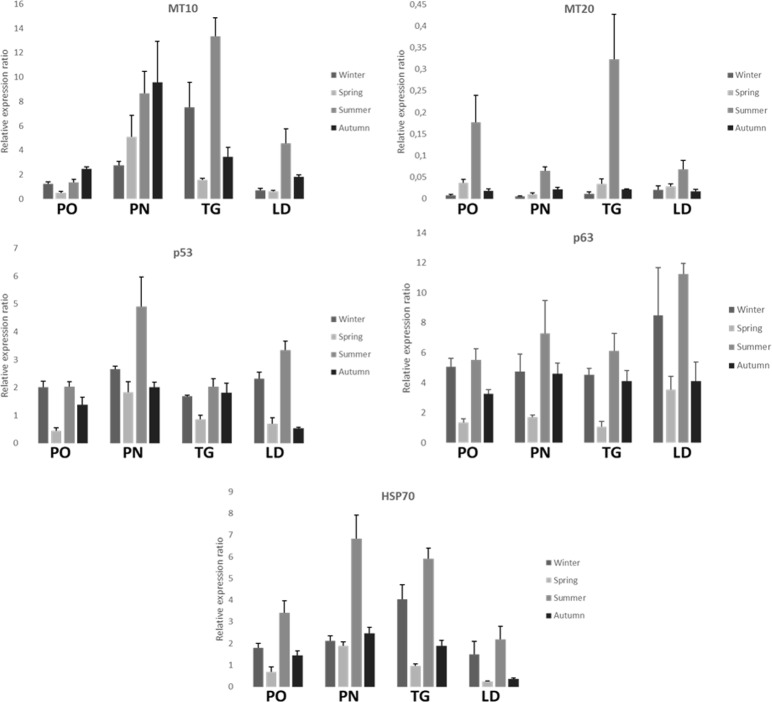
Relative expression ratio of *MT-10*, *MT-20 p53, p63*, and *HSP70* genes in the digestive gland tissue of *M. galloprovincialis* collected at the different sites and periods. Bars indicate the standard error.

**FIGURE 6:**
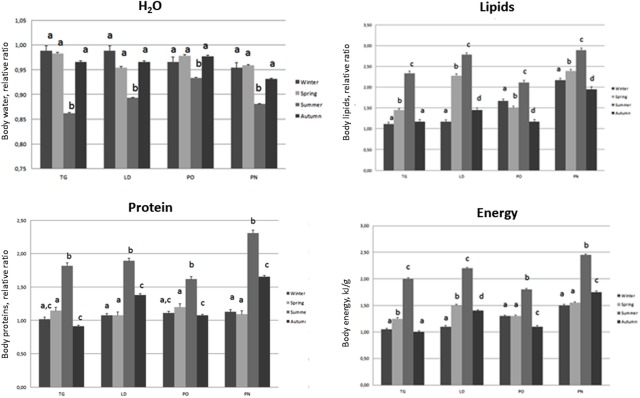
Body composition (Water, Lipid, Protein, Energy) of *M. galloprovincialis* collected at the different sites and periods. Different superscripted letters indicate statistically significant differences (*P* < 0.05).

**FIGURE 7:**
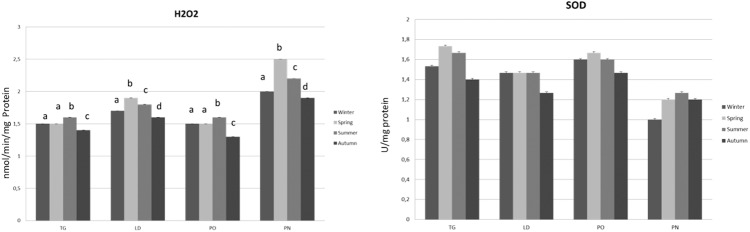
Hydrogen peroxide (H_2_O_2_) and SOD (Superoxide Dismutase) of *M. galloprovincialis* collected at the different sites and periods. Different superscripted letters indicate statistically significant differences (*P* < 0.05).

**FIGURE 8:**
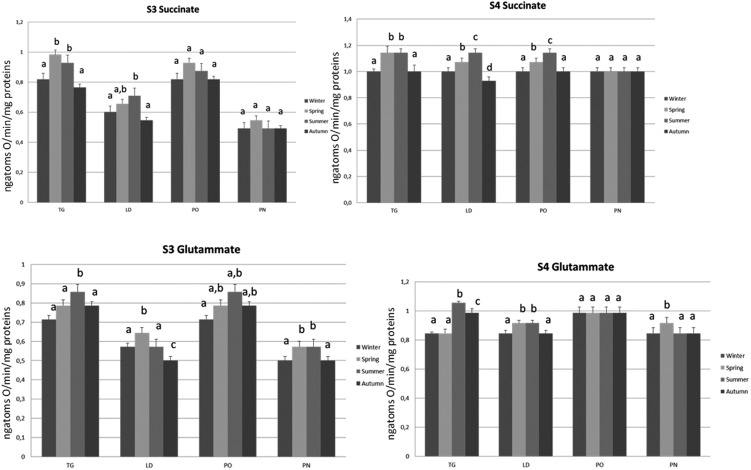
Mitochondrial respiration rates and oxidative stress of *M. galloprovincialis* collected at the different sites and periods. Different superscripted letters indicate statistically significant differences (*P* < 0.05).

**FIGURE 9:**
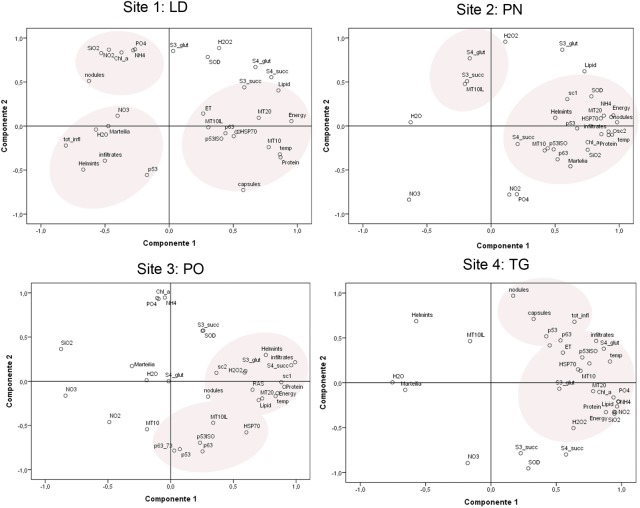
Principal component analysis and site groupings showing the relationship between mussel biomarker responses in the considered areas.

**Table 3:** Prevalence of the overall Pathogens and Symbionts recorded in *M. galloprovincialis* samples.

	Prokaryote	Protozoa			Metazoa		
Sample	*Nocardia crassostreae*	Cercozoa	Microsporidia	Gill Ciliated	Trematodes		Idrozoa
		
		*M. refringens*	*S. mytilovum*	*A. mytili*	*Urostoma* sp.	Trematodes Metacercariae/ Sporocysts	*E. inquilina*
LDa	0	3.3	10	0	0	0	3.3
LDb	0	0	0	6.6	3.3	0	0
LDc	0	10	10	0	0	10	0
LDd	0	10	3.3	0	10	6.6	13
POa	0	0	6.6	13	0	0	0
POb	0	0	10	0	0	0	0
POc	0	0	0	0	0	13	0
POd	0	0	0	13	0	0	0
PNa	6.6	3.3	10	0	0	6.6	6.6
PNb	13	23	6.6	0	0	0	0
PNc	3.3	6.6	0	3.3	0	6.6	0
PNd	3.3	16	0	6.6	0	0	0
TGa	0	6.6	16.6	3.3	3.3	10	26.6
TGb	0	33.3	0	0	0	16	6.6
TGc	0	26.6	0	3.3	0	0	3.3
TGd	0	0	0	0	0	23	6.6

**Table 4:** Environmental data in surface water of each area: inorganic nutrients [DIN ([NO_3_^-^ + NO_2_^-^ + NH4+), PO_4_^3-^, Si(OH)_4_] (μM); DIN/P ratio; Chl-a (μg L^-1^) and Phaeo/Chl-a ratio [Phaeopigments/Chl-a]; temperature (°C).

		DIN	PO_4_^3-^	Si(OH)_4_	Chl-a	Phaeo/Chl-a	Temp
LD	01/03/2012	6.665	0.576	5.501	2.175	0.67	14.40
	15/05/2012	13.773	3.839	7.806	5.914	0.48	18.13
	12/07/2012	0.313	0.076	1.001	1.012	1.01	27.80
	11/10/2012	0.924	0.081	1.264	1.516	0.52	22.15
PO	01/03/2012	1.108	0.044	1.273	0.459	0.66	14.34
	15/05/2012	0.894	0.116	1.322	2.191	0.33	17.92
	12/07/2012	0.276	0.029	0.493	0.134	0.52	27.91
	11/10/2012	0.349	0.031	1.135	0.276	0.34	22.71
PN	12/12/2011	30.730	2.210	3.750	6.450	0.74	17.10
	17/05/2012	13.561	0.635	7.894	1.757	1.20	18.38
	09/07/2012	80.889	3.881	17.206	16.030	0.52	27.88
	11/10/2012	41.838	10.381	18.356	6.435	1.47	23.69
TG	07/12/2011	3.180	0.270	1.620	3.160	0.62	16.90
	17/05/2012	84.636	0.468	45.025	2.262	0.51	18.09
	09/07/2012	36.103	2.017	161.131	10.470	0.88	27.29
	17/10/2010	5.576	0.298	6.746	1.463	0.96	22.95

**Table 5:** Pearson correlations between gene expression, inflammations, and environmental data.

	p53	1c p63	HSP70	tot_jnfl	temp	NH4	N02	N03	P04	Si02	Chl_a
p53	1	0.779**	0.702**	0.293	0.460	0.686**	0.300	-0.176	0.250	0.131	0.619*
		0.000	0.002	0.270	0.073	0.003	0.259	0.514	0.351	0.629	0.011
p63	0.779**	1	0.479	0.290	0.245	0.368	0.157	-0.247	0.109	0.104	0.370
	0.000		0.060	0.276	0.360	0.160	0.562	0.356	0.688	0.701	0.158
MDM	0.755**	0.813**	0.655**	0.532*	0.503*	0.523*	0.019	-0.355	0.024	0.155	0.465
	0.001	0.000	0.006	0.034	0.047	0.038	0.945	0.177	0.929	0.566	0.070
HSP70	0.702**	0.479	1	0.497	0.670**	0.484	0.090	-0.175	0.097	0.525*	0.556*
	0.002	0.060		0.050	0.005	0.057	0.740	0.516	0.720	0.037	0.025

*Significantly different values (<0.05^∗^, <0.01^∗∗^) are indicated in green.*

### Pathological Conditions Recorded

Specimens from the different areas showed diverse types of lesions, mostly represented by inflammations of various degree and type, and atrophy of epithelial tubules of digestive gland. In particular, inflammation such as haemocytic infiltration, nodules and capsules have been largely recorded in the digestive system of mussels, showing a temporal stratification, occasionally associated to protozoan parasites like *Marteilia refringens*, Fungi *Steinhausia mytilovum* and other pathogens (**Figures 1**–**4** and **Table 3**). Bacterial infections from *Nocardia crassostreae* was observed in the digestive gland of samples from PN area, with colonies presence always accompanied by a strong haemocytic incapsulation.

Regarding the digestive tissues, the histological integrity of the digestive gland tubules was reduced in mussels from most of the studied localities, mostly represented in all the period in PO and PN. In many cases the digestive tissue was occupied by a disorganized interstitial connective tissue with infiltrating haemocytes or occupied by granulocytomas. Digestive ducts could be dilated in some case containing lipofuscin and presented an enlarged lumen and thinnest epithelium (**Figures 2**–**3**). Necrotic tissue circumscribing adductor muscle was also recorded in LD (13%) and TG (10%) during winter season and macroscopically visible during sampling.

Interestingly, cases of Disorders of Sex Development (DSD) represented by intersex, were recorded during Spring and Summer in three of the examined areas at higher percentage (10–15%). Co-existence of both female and males follicles was observed, with mature oocytes and spermatozoa (**Figure 1E**).

### Gene Expression Analysis

**Figure 5** shows the relative expression levels of the target genes *MT-10*, *MT-20*, *p53*, *p63*, and *HSP70* of *M. galloprovincialis* in the different collection sites. Measures are reported as mean values ± standard error of the mean (SEM). All but one the genes (*MT-20*) show expression levels generally higher than the acclimated controls (not reported in the graphs, whose expression level is 1) during the time course of the samplings. All the examined genes show a peak of expression during the summer, and in some cases(e.g., *MT-10* for the PN sampling) the autumn. The PN and TG sites show the highest mean values of the biomarkers examined.

### Variations in Body Composition

Specimens from the different areas showed changes in body composition mostly linked to the sampling season. The most significant differences were observed during summer, when the organisms of the four different areas showed the lowest body water content associated with an increase in lipid, protein and energy content. A decrease of these parameters was disclosed during autumn and winter as reported in **Figure 6**.

### Mitochondrial Respiration Rates and Oxidative Stress

The influences of the seasonal variations on mitochondrial oxidative capacity was evaluated using FAD (succinate)- and NAD (glutamate)-linked substrates. The mitochondrial oxidative rates measured using succinate as substrate revealed that the specimens of TG and LD site displayed the lowest state 3 and state 4 values during autumn and winter, and a significant increase was observed during spring and summer. The organisms of PO area showed an increase in state 4 respiration rate during spring, further enhanced in summer season. No difference was noticed in the state 3 evaluation during the time course of samplings. Interestingly, no differences in mitochondrial respiration rates were observed in the specimens of PN site during the different times of the year and the values were significantly lower compared to the other sampling sites.

The mitochondrial respiration rates measured using glutamate/malate as substrates revealed that the specimens of TG site displayed a significantly higher state 3 and state 4 values during summer period as compared to the other seasonal sampling. The LD specimens showed an increase in state 4 respiration rate during spring and summer associated with the higher state 3 values in spring season as compared to the other periods. The lowest state 3 was found during autumn. The organisms of PO area showed no difference in the state 4 evaluation during the time course of samplings, whereas the highest state 3 rate was found in summer season. The organisms of PN site showed an increase in state 4 only during the spring period associated with highest values of state 3 in spring and summer seasons (**Figure 7**).

### Oxidative Stress

The organisms of PO and TG areas revealed a significant increase in H_2_O_2_ yield during summer season as compared to the other periods, the lowest production was found during autumn. The LD and PN specimens showed the highest H_2_O_2_ yield during spring season, whereas a progressive reduction were disclosed in the other times of the year (summer > winter > autumn).

The specimens of TG site showed the lowest SOD activity during autumn, an increase was noticed in winter and summer and the highest activity was revealed during the spring period. The SOD activity in the LD specimens results significantly reduced during autumn as compared to the other seasons. In the organisms of PO area, this enzymatic activity was reduced during autumn and increased in the other periods of the year, the highest SOD activity was found in spring. In the PN area a higher SOD activity was found in summer season as compared to the other sampling, the lowest activity was measured during the winter (**Figure 8**).

### Environmental Data

Dissolved inorganic nitrogen (DIN [NO_3_^-^ + NO_2_^-^ + NH_4_^+^]), PO_4_^3-^ and Si(OH)_4_ concentrations were high in the investigated areas (**Table 4**). At station PN, the highest concentrations of DIN and PO_4_ are detected. A marked difference is also evident among the samples collected on the site of TG and LD compared to the site of PO. On the overall, the DIN/P ratio (calculated from the slope of DIN plotted against PO_4_^3-^) shows an altered ratio in the sites. The DIN/P ratio has exceeded 16 during all seasons in PN, PG stations; on the contrary, in LD and PO stations (except in winter) the DIN/P ratio is much lower. High concentration of silicates was found in TG and PN stations.

Chlorophyll-a (Chl-a) are relatively high and reflected the accumulation of the phytoplankton biomass in the sampling sites (**Table 4**). The highest values of Chl-a were recorded during summer in the PN and TG stations, with values of 16.030 and 10.470 μg L^-1^, respectively (**Table 4**), while in the PO station the mean value is 0.765 μg L^-1^. The presence of degraded pigments (high values of Pheo/Chl-a ratio) were recorded above all in the PN station.

During the sampling period, the surface temperature reflects the seasonal sampling period and varied from 14.34 to 27.91°C (**Table 4**).

The PCA of the biomarker responses (**Figure 9**) confirmed the importance of these biomarkers in distinguishing the responses amongst the different sites. Several of the biomarkers showed significant correlations. In all the areas, different stress proteins are activated, showing to be linked to inflammatory lesions (infiltrative and nodular) and pathogens, also linked to organic input and temperature.

## Discussion

A variety of stressors may affect the life of an organism, ranging from pollutants of different nature to climate changes. The stress response is a defense reaction of cells to damage that environmental forces inflict on cells and tissues and that then rebound on the entire individual. Therefore, depending on the intensity and the source of stress, different protection mechanisms and prosurvival strategies are mounted. In monitoring programs, mussels are considered suitable organisms for environmental quality assessment since they are able to provide cellular and physiological responses to different stress condition but giving important information on environmental status (Garmendia et al., 2011).

In this paper, we assessed and discussed the seasonal responses of tissue and molecular biomarkers, also correlated with environmental data in mussels located in different coastal sites of Campania Region (Italy).

Principal component analysis (PCA) was applied, showing the strongest correlation linked to the areas other than to the sampling season.

The results of pathological data conducted on samples from the different sites underlined the presence of various pathogenic organisms and lesions of different type. In most of the cases, the prevalence of pathogens and pathologies varied throughout the year with differences only being observed within individual months. Many studies have focused on the digestive tubule epithelial characteristics as target organ for related to chemical exposure. In marine mussels, as in other bivalve species, the digestive gland is a central organ for animal digestion and homeostasis. Changes in morphology of digestive tubules, along with other histopathological alterations, represent a non-adaptive response to pollutant exposure and stress (Cajaraville et al., 1992; Marigómez et al., 1992, 1996; Carella et al., 2015b). Atrophy and changes in the morphology of the digestive alveoli constitute a non-specific, fast inducible and slowly/not recoverable response to stressful environmental conditions (Brown et al., 1992). Histopathological changes in the digestive structure bring consequently to impairment functions of digestive activity and consequent modification of animal physiology. Previous studies have reported increased atrophic condition of the digestive gland epithelium following both short- and long-term exposure to PAHs or other pollutants. In our study, regressive changes at digestive tissue level were frequently reported. The digestive gland tissue showed the most severe damage in the mussels from LD and PN over the samplings; the most represented lesions were lumen dilation, and tubule necrosis correlated with inflammatory lesions, as also previously reported in the bivalves from heavily stressful and polluted sites (Syasina et al., 1997; Usheva et al., 2006).

Depending on the host age and stress conditions, environmental factors such as temperature, oxygen, nutrient presence and other factors can cooperate to activate pathogen virulence in susceptible species, thus triggering parasite/pathogen replication and transmission (Harvell et al., 1999). About the presence of the OIE listed *Marteilia* sp. pathogen and the microsporidia *S. mytilovum*, these parasites are considered potentially harmful, but mussel populations of different areas have shown distinct vulnerability related to their genetic origin (Bignell et al., 2008). Although the presence of *Marteilia* is not always harmful in this specific bivalve species, environmental conditions like high water temperatures for continued periods or low water quality, can weaken the animal, leading to disease or death of the most vulnerable animals (Audemard et al., 2001). Previous works reported the presence of *Marteilia refringens* in *M. galloprovincialis* farms in the Gulf of Naples (Carella et al., 2010); however, in the present study the authors detected higher prevalence both in the farm and in the wild population. Recent studies revealed that, at least in Mediterranean waters, where temperatures reach high values, *Marteilia* parasite has two periods for optimal multiplication and transmission during the year: spring/summer and autumn. This dynamic is correlated with bivalve spawning, zooplankton distribution in the area, also linked to food abundance and temperatures (Boyer et al., 2013). The bacteria belonging to *Nocardia* sp. normally exist as soil saprophytes, ubiquitous in fresh and salt water, dust, decaying vegetation and fecal deposits from animals. Since the late 1940s, nocardiosis of bivalves has been observed in association with summer mortality in Pacific oysters at several warm locations along the coasts of Japan and subsequently North America (Takeuchi et al., 1955), also reported in the Gulf of Naples in flat oysters and Mediterranean mussels (Carella et al., 2013a). Infection and mortalities seem to be highest for animals lives on a muddy substrate. However, other environmental factors such as reduced water circulation of superficial embayments, warmest temperatures and great nutrient levels can also increase the prevalence of infection of nocardiosis (Bower et al., 2005) as also reported in the PN station as showed by Chl-a values. Interestingly, recent reports assessed for the first time infection of *N. crassostreae* as emerging human pathogen causing invasive pulmonary nocardiosis in immunosuppressed and immunocompetent patients (Taj-Aldeen et al., 2013; Igbaseimokumo et al., 2016). This data suggest *N. crassostreae* to be a possible emerging disease with consequent possible risk of zoonotic infections. The DSD, Disorders of Sex Development, is a condition in which development of chromosomal, gonadal or anatomical sex is atypical and the reproductive organs differ from those classically associated as being male or female (Kim and Kim, 2012). This condition has been reported in humans and in wild animals like bird, fishes and reptiles, and related with specific types of pollutants like endocrine disrupting chemicals (EDC) (Khan and Law, 2005). The EDC interfere with animals hormonal systems, such as androgen and estrogen signaling pathways and resulting in an increased frequency of failure of the reproductive organs bring to lowered fertility (Schug et al., 2011). In estuarine/marine environments, effects on fish reproduction with up-regulation of aromatases and vitellogenins in males and juveniles and the presence of intersex individuals was reported (Ortiz-Zarragoitia et al., 2014). In bivalves, growing evidence supports the hypothesis that a wide range of EDC can affect sex determination, sex ratio, gonadal and gamete development, and larval growth (Nice et al., 2003; Nice, 2005). The mussel *M. galloprovincialis* is a strictly gonochoristic species (Gosling, 2003). Lately, cases of abnormal hermaphroditism were reported in literature in dioecious species such as *Mytilus edulis* and *M. galloprovincialis* (Devauchelle, 1990; Ponurovsky and Yakovlev, 1992) and related to this specific kind of chemicals. In this study, interestingly, mussels of autumn season from LD and TG and in summer from PN showed signs of distressed gamete development, with high prevalence of oocyte atresia linked to high incidence of hermaphroditism/intersex cases.

In mussels, metallothionein belongs to two different gene classes, MT10 and MT20, reported to show different expression at basal conditions and under heavy metal challenge (Aceto et al., 2011). At the same time, heat shock proteins (HSPs) chaperone activity regulates the activity of proteins that are involved in cell cycle machinery like p53, that has an important role in cell cycle control and apoptosis (Zylicz et al., 2001). Under stress condition, like heat shock, inhibitors of energy metabolism, presence of heavy metals, oxidative stress or inflammation, HSP70 increase expression bring to an increased cell survival strategies by protecting and disaggregating stress labile proteins (Skowyra et al., 1990), and with the proteolysis of the damaged proteins (Wickner et al., 1999). In our study, inflammatory and DSD lesions form these areas showed a strong correlation with the expression levels of the *p53*, *MT-10*, *MT-20*, *HSP70* genes and high inorganic nutrient input. On the other side, presence of infiltrative and nodular inflammations was also linked to *MT-10*, *MT-20* expression levels, but mostly with higher temperature, SOD and NH_4_. The high level of expression of the *p53*, *MT-10*, and *HSP70* genes in the mussels collected in the sites examined, in particular PN and TG, suggests that these animals are subjected to a general environmental stress possibly due to the scarce quality of the water for an increase in the supply of organic matter in these sites.

At the same time, the highest values of Chl-a were recorded in the PN and TG stations (16.030 and 10.470 mg L^-1^, respectively) and reflected the phytoplankton biomass due to effects of anthropogenic nutrient loading. In PN station, the degraded pigments (high values of Pheo/Chl-a ratio) were recorded. The high Pheao/Chl-a ratios suggest the occurrence senescent (instead of grazed) phytoplankton cells (Hendry et al., 1987). This aspect suggests the accumulation of phaeopigments within phytoplankton cells during adverse growth conditions (Hendry et al., 1987) and/or pheopigments packed in fecal pellets with could be rapidly exported from surface waters to the benthic community bottom, (Caron et al., 2004) constituting an important food source for benthic community (Caron et al., 2004). Differently, in the PO station the mean value of Chl-a is 0.765 mg L^-1^ that fall within the range of those found for the Gulf of Naples and for coastal areas with low nutrient enrichment (Ribera d’Alcalà et al., 2003; Mangoni et al., 2016). The DIN/P ratio shows an altered ratio due to local inputs or to a high uptake of nitrogen by phytoplankton. The DIN/P ratio has exceeded 16 during all seasons in PN, TG stations, thus marking a phosphorous limitation; while in LD station and PO station the DIN/P ratio is much lower to indicate a likely nitrogen-limitation (Ribera d’Alcalà et al., 2003; Pujo-Pay et al., 2011). TG station is densely populated areas, characterized by the presence of many industrial and port activities. During the years, this condition caused widespread contamination of marine sediments, leading to progressive accumulation and pollution of water, air and land (Imperato et al., 2003).

Since the activity of antioxidant enzymes and H_2_O_2_ yield are known to be under extensive seasonal control (Sheehan and Power, 1999), the seasonal pattern of antioxidant defense enzymes found in *M. galloprovincialis* may be associated to the seasonal variations of temperature and the reproductive cycle. In the mussel *Modiolus modiolus* it was demonstrated by an increase of the concentration of rate-limiting metabolic enzymes to compensate the reduction of the temperature, while the same level of antioxidant protection were maintained in Summer and Winter (Lesser and Kruse, 2004).

Furthermore, the increase in temperature is followed by an increase in oxygen consumption and by an increase in ROS generation. Also the metabolic particularity of the stages of the reproductive cycle represents a season-related determining factor and influences the levels of antioxidants in the mussels. Therefore, the obtained results and the conclusions of this study reinforce the importance of seasonality on the antioxidant status of Bivalvia in relation to the interpretation of biomonitoring data (Wilhelm-Filho et al., 2001).

## Conclusion

Cellular stress response is part of the normal physiology of every animal ensure cell survival.

In this study tissue responses provided insight into the mussel health status of the area. Histopathology has been deeply used in studies of aquatic animal health, coupled with other tools common in different monitoring programs. The integration of multiple histopathological traits along with marker of cellular stress permitted a more sensitive discrimination of different sites and provided a ranking of sites that is in agreement with environmental variables. A series of pathogens and lesions were observed, one of them also of interest also for human health. The most diverse community of parasites and lesions linked to stressors was observed at sites with poor water quality. The major areas of concern, the PN and PO, were observed to be compatible with respect to environmental quality. Further research are in course in our laboratory to clarify possible causes of the observed effects in mussels from Campanian coastline.

## Author Contributions

FC: pathological study and organization of the work. SA: gene expression analysis. OM: environmental data analysis. MM: stress response and energy content. GC and GT: stress response and energy content. FA: bioinformatic analysis. GDV: pathological study.

## Conflict of Interest Statement

The authors declare that the research was conducted in the absence of any commercial or financial relationships that could be construed as a potential conflict of interest.
